# Novel biomarkers of inflammation-associated immunity in cervical cancer

**DOI:** 10.3389/fonc.2024.1351736

**Published:** 2024-03-12

**Authors:** Weihong Zhao, Qi Li, Songquan Wen, Yaqin Li, Ying Bai, Zhiyu Tian

**Affiliations:** ^1^ Department of Obstetrics and Gynecology, The Second Hospital of Shanxi Medical University, Taiyuan, Shanxi, China; ^2^ The Second Clinical Medical College, Shanxi Medical University, Taiyuan, China; ^3^ Department of Obstetrics and Gynecology, Peking University People’s Hospital, Beijing, China; ^4^ Department of Epidemiology, School of Public Health, Shanxi Medical University, Taiyuan, Shanxi, China

**Keywords:** cervical cancer, immune infiltration, differentially expressed inflammation-related genes, inflammation-associated immune biomarkers, CIBERSORT

## Abstract

**Background:**

Cervical cancer (CC) is a highly malignant gynecological cancer with a direct causal link to inflammation, primarily resulting from persistent high-risk human papillomavirus (HPV) infection. Given the challenges in early detection and mid to late-stage treatment, our research aims to identify inflammation-associated immune biomarkers in CC.

**Methods:**

Using a bioinformatics approach combined with experimental validation, we integrated two CC datasets (GSE39001 and GSE63514) in the Gene Expression Omnibus (GEO) to eliminate batch effects. Immune-related inflammation differentially expressed genes (DGEs) were obtained by R language identification.

**Results:**

This analysis identified 37 inflammation-related DEGs. Subsequently, we discussed the different levels of immune infiltration between CC cases and controls. Weighted gene co-expression network analysis (WGCNA) identified seven immune infiltration-related modules in CC. We identified 15 immune DEGs associated with inflammation at the intersection of these findings. In addition, we constructed a protein interaction network using the String database and screened five hub genes using "CytoHubba": CXC chemokine ligand 8 (CXCL8), CXC chemokine ligand 10 (CXCL10), CX3C chemokine receptor 1 (CX3CR1), Fc gamma receptors 3B (FCGR3B), and SELL. The expression of these five genes in CC was determined by PCR experiments. In addition, we assessed their diagnostic value and further analyzed the association of immune cells with them.

**Conclusions:**

Five inflammation- and immune-related genes were identified, aiming to provide new directions for early diagnosis and mid to late-stage treatment of CC from multiple perspectives.

## 1 Introduction

Cervical cancer (CC) is one of the most frequent malignant tumors in women, imposing a substantial burden on patients, their families, and society at large. In 2020, the number of new cases and deaths from cervical cancer will remain persistently high globally, with China reporting the highest incidence and mortality rates for CC (1). Despite noteworthy advancements in the diagnosis and management of CC in the last few years, the results remain unsatisfactory. Presently, colposcopy and cervical biopsy are the main diagnostic methods for CC (2). These techniques, however, rely heavily on subjective judgement, resulting in a low penetration rate, poor diagnostic sensitivity and specificity, and reduced predictive efficiency. This, in turn, contributes to an annual growth in the number of CC cases, particularly among younger age groups. Early stage CC can be managed surgically and has a good prognosis, while advanced CC has a significantly worse prognosis (3). Therefore, it is important to prioritize early diagnosis and adjuvant treatment.

CC is primarily caused by chronic inflammation, and its main causative factor is persistent infection with high-risk types of human papillomavirus (HPV). HPV, a spherical DNA virus, targets epithelial cells, disrupting the normal cell cycle and promoting cytogenetic damage, resulting in abnormal cell division (4). The immune system in most cases effectively clears HPV infections, with only 1% of cases progressing to CC (5). Therefore, the development of CC is related to HPV infection as well as to the immune system, which has a major impact on immune surveillance and clearance (6). The human immune system has been shown to be a determinant of cancer development and progression, with immune cells such as Immune cells such as macrophages, B cells, natural killer cells, and dendritic cells being key players in the process (7). Furthermore, the type, distribution, and degree of infiltration of immune cells within different tumors vary significantly (8). Moreover, many reports have demonstrated that immune cell infiltration is critical in the development, progression, and treatment of CC (9–11). However, there are no reports that have studied the combination of inflammation and immune cell infiltration in CC. Thus, we identified five inflammation and immune infiltration related biomarkers including CXC chemokine ligand 8 (CXCL8), CXC chemokine ligand 10 (CXCL10), CX3C chemokine receptor 1 (CX3CR1), Fc gamma receptors 3B (FCGR3B) and SELL. These findings may contribute to the early detection and treatment of CC, and are valuable for establishing non-invasive diagnostic methods, identifying new therapeutic targets and elucidating the mechanistic studies of CC development.

## 2 Methods

### 2.1 Data processing and screening of differentially expressed genes

Gene expression microarrays of CC were obtained in the Gene Expression Omnibus (GEO) database (https://www.ncbi.nlm.nih.gov/geo/), including GSE39001 (CC = 28, control = 24) and GSE63514 (CC = 43, control = 12). These datasets were normalized to the data at the time of upload. When multiple probes corresponded to the same gene, expression values were averaged. To eliminate batch effects and other unwanted variations between the two datasets, the bioconductor Substitute Variable Analysis (SVA) package was used (12). The processed data were screened for DEGs through the “limma” package, with screening criteria set at P.adjust < 0.05 and |log2FC|>1 (Fold Change). Subsequently, gene heat maps and volcano plots were drawn using the R software, with volcano plots using log2FC as the horizontal coordinate and log10 (P.adj) as the vertical coordinate.

### 2.2 Identification of inflammation-related DEGs

We used the GeneCards database to identify 320 inflammation-related genes with correlations greater than 6. To categorize them for further analysis, we utilized the “VennDiagram” R package, intersecting these genes with DEGs associated with inflammation in order to define them as inflammation-related DEGs.

### 2.3 GO and KEGG analysis

We performed GO functional and KEGG pathway enrichment analyses. GO functional enrichment analyses included Biological Process (BP), Cellular Component (CC) and Molecular Function (MC). P<0.05 indicates that the results are significantly differentially enriched, which can be visualized using the R software ggplot2 package.

### 2.4 Immune cells infiltration correlation analysis

Using standardized gene expression patterns, CIBERSORT is capable of quantifying the relative proportion of infiltrating immune cells in a given sample. By downloading the corresponding data from the CIBERSORT website (http://CIBERSORT.stanford.edu/) and performing CIBERSORT analyses to merge the expression data, we can calculate immune cell infiltration (13). We use a threshold of p < 0.05 to filter the samples. We calculated the percentage of each immune cell type in each sample and analyzed the association between the number of 22 immune cells and new genes. The corplot and vioplot packages in R were used to visualize the result.

### 2.5 Weighted gene co-expression network analysis

We employed WGCNA to investigate gene interactions. Firstly, we imported processed data into WGCNA, then eliminated outlier samples to enhance the accuracy and reliability of the network construction results. Following this, We construct the scale-free network using the function “pick soft threshold”, selecting a soft power of β = 2. Then we transform the adjacency matrix into a topological overlap matrix (TOM) and compute the dissimilarity (1-TOM). Fourth, the functional detection module was extracted using hierarchical clustering and a dynamic tree. To classify genes with similar expression profiles as gene modules, average-chained hierarchical clustering was performed based on a TOM-based dissimilarity measure; the minimum size of the gene dendrogram to 60 (genome) was set. Finally, the WGCNA software package can be used to calculate correlations between differentially infiltrating immune cells and gene modules. We can identify candidate modules that are related to differential infiltration of immune cells through calculating correlation coefficients. Immune-related inflammation differentially expressed genes (Immune-related inflammation DGE) were further identified by plotting a Wayne diagram visualizing the intersection of immune-related differentially expressed genes (Inflammation-related DEGs) with genes in the significant modules.

### 2.6 PPI network construction and screening of key genes

The STRING database (https://www.string-db.org/) enabled us to build PPI networks of target genes. The screening conditions were a reciprocal score of > 0.4. The PPI network can be visualized using Cytoscape software (http://www.cytoscape.org/). With the help of the cytoHubba plugin in Cytoscape, we were able to screen the five most important genes (hub genes) using the MCC algorithm.

### 2.7 Construction and prediction of regulatory networks of potential TF and miRNA target genes

The miRNet online database (https://www.mirnet.ca/) was used to find out possible miRNA target diagnostic genes and to predict upstream transcription factors (TFs). To visualize these results, Cytoscape software can be used.

### 2.8 ROC curve analysis and expression analysis

The ‘pROC’ package was employed to plot Receiver Operating Characteristic (ROC) curves to validate the accuracy of each hub gene diagnosis (14). Hub genes with AUC>0.7 were considered important for disease diagnosis.

### 2.9 Correlation analysis of infiltrating immune cells with diagnostic genes

We performed Spearman correlation analysis between infiltrating immune cells and diagnostic genes based on the above immune infiltration results. Also to show the correlation more intuitively, we used visualization tools to generate lollipop plots showing the association between diagnostic genes and immune cells. The above process was implemented by corrplot and ggpubr packages in R.

### 2.10 PCR validation of expression

In this study, we collected tissue samples from those who underwent colposcopy between January and April 2023 for abnormal CC screening. Inclusion criteria were: single HPV16 infection; local residence for at least one year; and none were pregnant, had not undergone hysterectomy and treatment for cervical or vaginal lesions, and had no history of other malignancies. By rigorously matching the epidemiological data, including age ( ± 2 years), number of pregnancies, number of births, and number of sexual partners, we finally selected 5 cases of chronic cervicitis and 6 cases of CC tissue with a clear pathological diagnosis for the validation study of the gene. The clinical stages of CC in this group were all ≤ stage IIa1. Written informed consent was obtained from all participants and the study was also approved by the Ethics Review Committee of the Second Hospital of Shanxi Medical University [IRB no. (2019) YX (280)].

We examined the expression of target gene in HPV16-positive normal and cervical cancer tissues using real-time PCR reverse transcription methods under suitable conditions. Each group contained 5-6 samples. The real-time fluorescence quantitative PCR experimental experiment consisted of the following steps: firstly, a pre-denaturation of 30 seconds at 95°C; followed by 40 cycles of PCR reaction, each cycle lasting 3 seconds and still at 95°C; and finally 30 seconds at 60°C. Cycling threshold (Ct) values were recorded, and the 2-ΔΔCt function was applied to compute target gene expression. To accurately measure the relative amount of change in gene expression, we normalized the data using 18S rRNA or GAPDH as internal reference standards. Please refer to **Table 1** for information on specific primer sequences.

**Table 1:** Primers for PCR assay.

Name	Primer sequence
CXCL8-F	CTCTTGGCAGCCTTCCTGATTTC
CXCL8-R	GGGTGGAAAGGTTTGGAGTATGTC
CXCL10-F	AGGGTGAGAAGAGATGTCTGAATCC
CXCL10-R	AGACCTTTCCTTGCTAACTGCTTTC
CX3CR1-F	CCTGTCCATATTCTACTCCGTCATC
CX3CR1-R	GGCTTCTTGCTGTTGGTGAGG
FCGR3B-F	GCGTGCTTGAGAAGGACAGTG
FCGR3B-R	TGTGGCAGCGTCAATGAAGTAG
SELL-F	ACAACAAGAAGAACAAGGAGGACTG
SELL-R	TGGCAGGCGTCATCGTTCC

## 3 Results

### 3.1 Identification and functional enrichment analysis of inflammation-related DEGs

**Figure 1:**
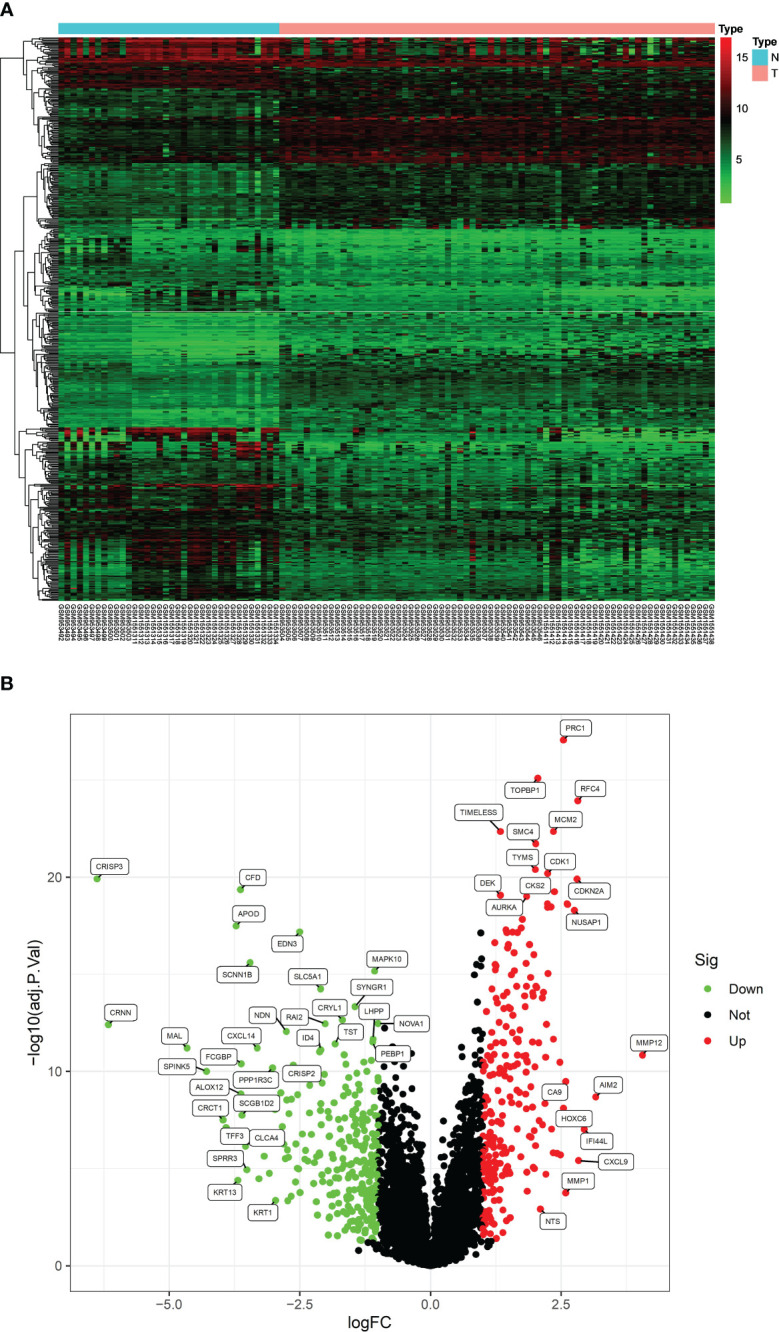
Thermogram **(A)** and volcano **(B)** diagram for identification of DEGs.

**Figure 2:**
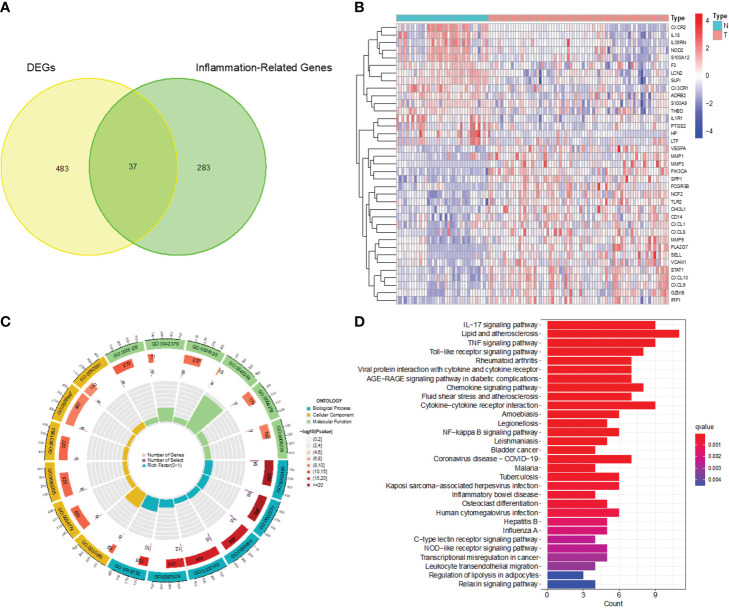
Identification of inflammation-related DEGs and functional enrichment analysis. **(A)** Venn diagram of DEGs and inflammation-related genes. **(B)** Heatmap for identification of DEGs. **(C)** GO analysis of inflammation-related DEGs. **(D)** KEGG analysis of inflammation-related DEGs.

After analyzing the processed data, we detected 520 differentially expressed genes (DEGs), comprising 240 upregulated and 280 downregulated genes (**Figures 1A, B**). These screened DEGs were further intersected with inflammation-related genes to identify 37 inflammation-related DEGs (**Figures 2A, B**). Subsequently, we conducted GO and KEGG enrichment analyses on the obtained 37 inflammation-related DEGs to explore the biological functions related to inflammation in CC (**Figures 2C, D**). In the GO enrichment analyses (see **Figure 2C**), we found that these genes were associated with various processes, including response to lipopolysaccharide, specific granule lumen, and cytokine receptor binding. For the KEGG enrichment analysis (**Figure 2D**), our findings revealed associations with pathways such as the IL-17 signaling pathway, lipid metabolism, and TNF signaling.

### 3.2 Analysis of immune infiltration

**Figure 3:**
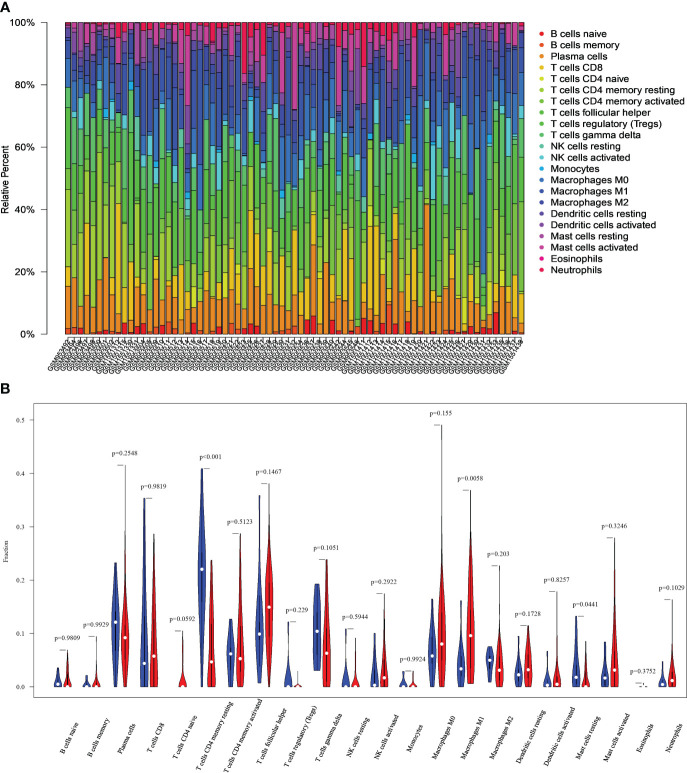
Differences in immune characteristics between normal and CC tissues. **(A)** Relative percentages of 22 immune cells in normal and CC tissues. **(B)** Comparison of infiltrating immune cells between normal and CC tissues.

**Figures 3A, B** show the immune cell profiles for 22 distinct types identified in CC and normal tissues. **Figure 3A** shows the proportions of infiltrating immune cells within each sample. Based on the data in **Figure 3B**, we can find significant differences (P<0.05) between CC and normal tissues (P<0.05) in the 3 immune cell types. These include resting mast cells, M1 macrophages, and CD4+ memory resting T cells. Specifically, the CC group exhibited increased macrophages M1, as well as decreased mast cells resting and T cells CD4 memory resting compared to the control group.

### 3.3 Construction of WGCNA immune-expression network and acquisition of key modules

**Figure 4:**
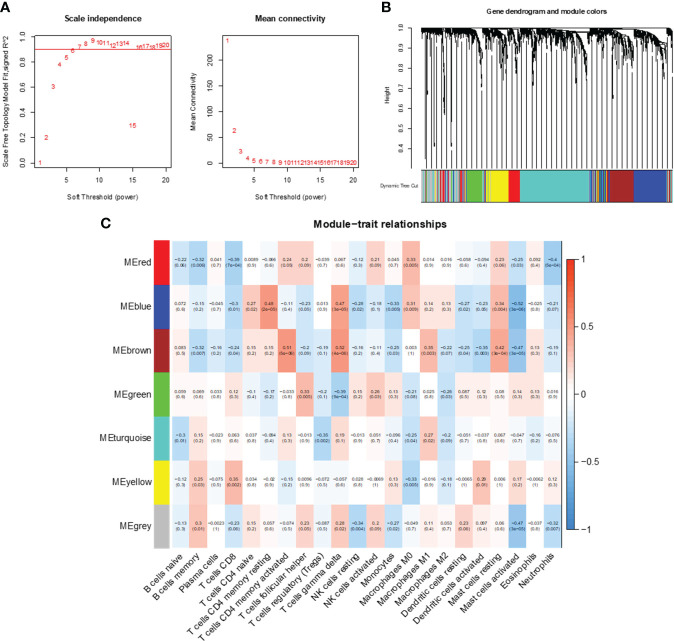
Co-expression network analysis of immune-related genes. **(A)** Optimal soft threshold power. **(B)** Immune-related co-expressed gene modules indicated by different colors under the gene tree. **(C)** Heatmap of association between WGCNA modules and immune cells.

WGCNA was used to analyze gene expression correlations, resulting in 1445 genes showing significant correlations with each other. A soft threshold of β = 6 was set for gene expression correlation, and a weighted gene co-expression network was exhibited (**Figure 4A**). Subsequently, we conducted cluster analysis with a minimum module size set to 60, generating distinct gene modules and hierarchical clustering trees. These trees were then clipped with a similarity coefficient of 0.25, resulting in seven gene modules (**Figures 4B, C**). Notably, among these modules, MEblue and MEbrown showed the strongest correlation with features (immune cell infiltration in CC). The blue module was highly negatively correlated with activated mast cells, and the brown module was highly positively correlated with CD4+ memory T cells and gamma delta T cells. Therefore, we determined the close correlation of these two modules with immune infiltrating cells and analyzed them further.

### 3.4 Identification and functional enrichment analysis of immune-associated inflammation DEGs

**Figure 5:**
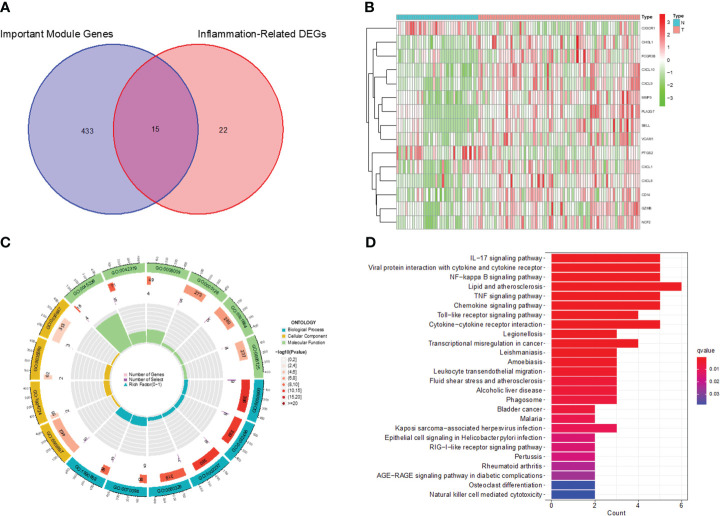
Identification of Immune-associated inflammation DEGs and functional enrichment analysis. **(A)** Venn diagram of inflammation-associated DEGs and important modular genes. **(B)** Heatmap for identification of Immune-associated inflammation DEGs. **(C)** GO analysis of Immune-associated inflammation DEGs. **(D)** KEGG analysis of Immune-associated inflammation DEGs.

We screened the Inflammation-Related DEGs intersecting with important modular gene fetches to obtain 15 immune-associated inflammatory DEGs (**Figures 5A, B**). We analyzed these genes for KEGG pathway enrichment and GO annotation (**Figures 5C, D**). The GO analysis showed that these 15 genes were mainly enriched in processes such as leukocyte migration, the external side of the plasma membrane, and CXCR chemokines. In addition, the KEGG analysis confirmed their enrichment in pathways such as the IL-17 signaling pathway, viral protein interaction with cytokines and cytokine receptors, and NF-kappa B signaling pathway, etc.

### 3.5 Construction of protein interactions network and screening of hub genes

**Figure 6:**
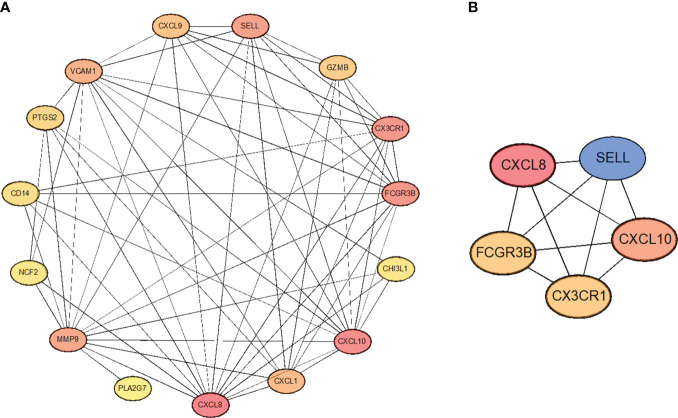
**(A)** Protein-protein interaction network. **(B)** Hub genes extracted from the PPI network.

In order to select the most important core genes from the above 15 genes, we used the String database to construct protein interaction networks and used Cytoscape software to present the results. (**Figure 6A**). Simultaneously, we used the Cytoscape package “CytoHubba” to screen five hub genes in the center of the interaction network, including CXCL8, CXCL10, CX3CR1, FCGR3B, and SELL (**Figure 6B**). In addition, correlations between these five hub genes are shown. These five genes are the core of the protein interaction network; along with their expression varying in tumor cells, they also interact with most other differentially expressed genes.

### 3.6 miRNA-TF-mRNA regulatory network

**Figure 7:**
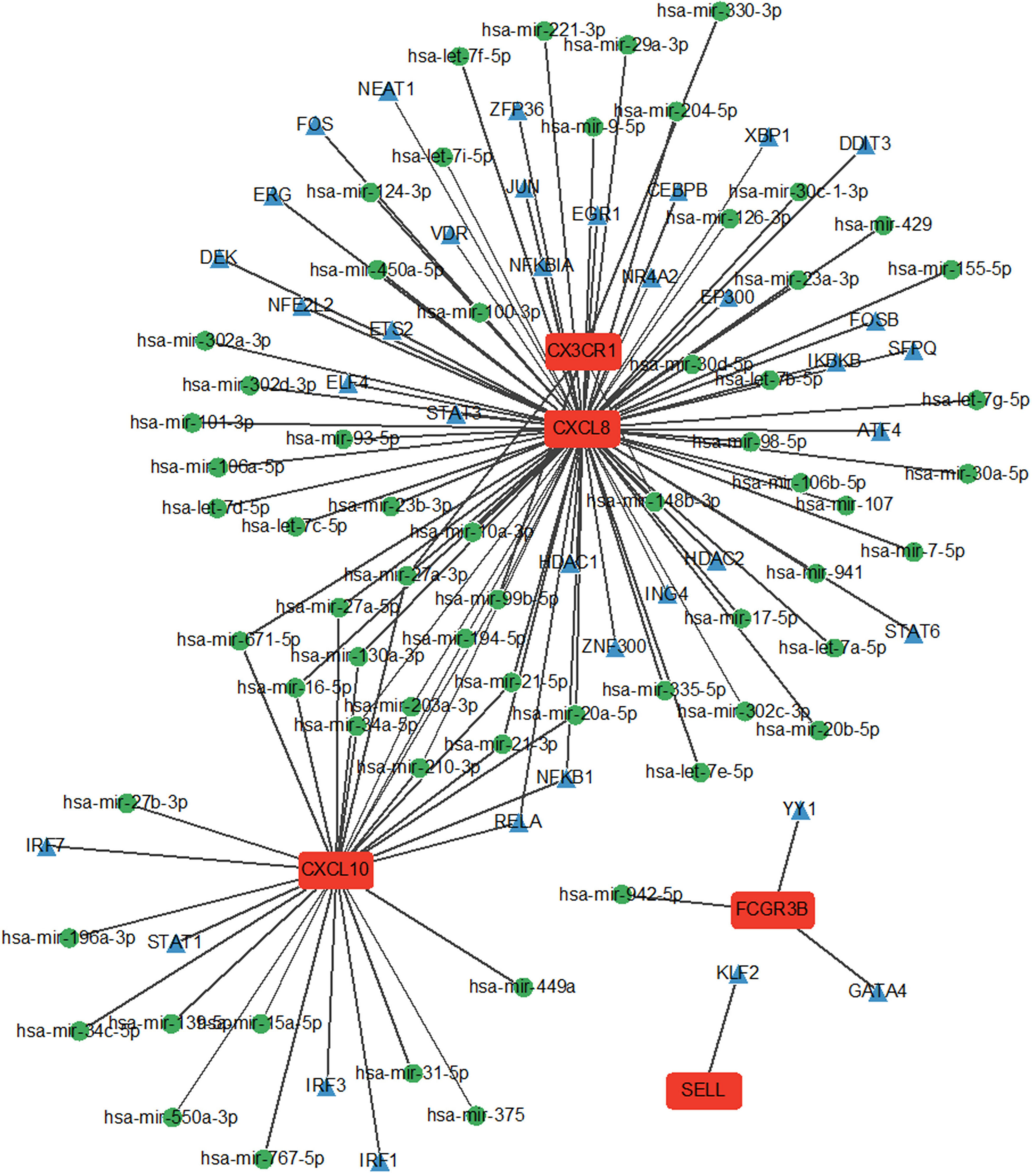
miRNA network and TF network of Hub genes. Green nodes represent miRNAs and blue nodes represent TFs.

The interaction network comprised five Hub genes, 80 miRNAs, and 39 TFs (**Figure 7**). Among these, 29 TFs, including ATF4, CEBPB, and DDIT3, regulate CXCL8 expression. Six TFs: IRF1, IRF3, IRF7, NFKB1, RELA, and STAT1 regulate CXCL10 expression. Two TFs, GATA4 and YY1, regulate FCGR3B expression, while SELL is regulated by one TF and KLF2.

### 3.7 ROC curve analysis of hub genes

**Figure 8:**
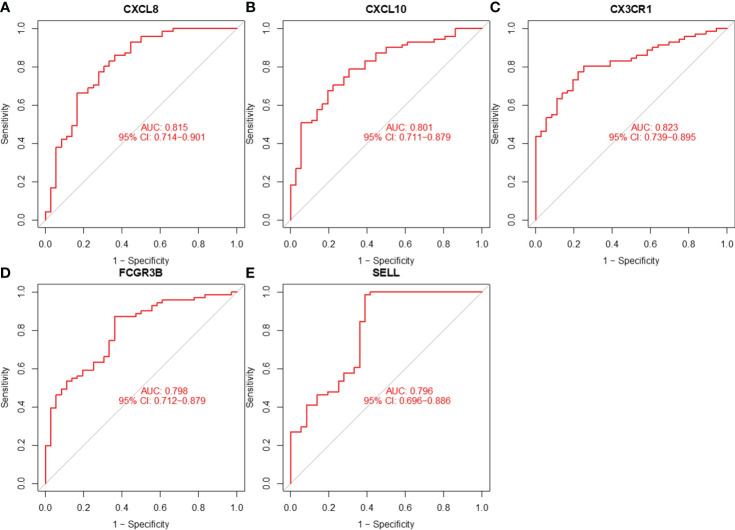
ROC curve analysis of **(A)** CXCL8, **(B)** CXCL10, **(C)** CX3CR1, **(D)** FCGR3B, and **(E)** SELL.

We assessed the value of five hub genes in the diagnosis of CC by plotting ROC curves. (**Figure 8**). The five hub genes (AUC>0.7) could be used as diagnostic markers. Specifically, the AUCs of these genes were 0.815, 0.801, 0.823, 0.798, and 0.796, signifying their substantial diagnostic value.

### 3.8 Correlation analysis between hub genes and immune cells

**Figure 9:**
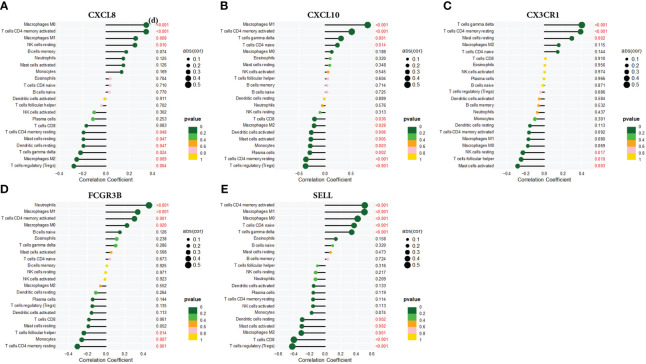
Correlation of **(A)** CXCL8, **(B)** CXCL10, **(C)** CX3CR1, **(D)** FCGR3B, and **(E)** SELL with infiltrating immune cells.

Using the CIBERSORT technique,we investigated the correlation between CXCL8, CXCL10, CX3CR1, FCGR3B, and SELL expression levels and infiltrating immune cells in CC. Specifically, CXCL8 exhibited strong positive correlations with Macrophages M0 and CD4 memory-activated T cells, while displaying pronounced negative correlations with regulatory T cells (Tregs). CXCL10 showed positive correlations with Macrophages M1 and negative correlations with Tregs. CX3CR1 displayed positive correlations with T cell gamma delta and highly negative correlations with activated Mast cells. FCGR3B showed positive correlations with neutrophils and marked negative associations with CD4+ memory resting T cells. SELL exhibited pronounced positive correlations with CD4+ memory-activated T cells and Macrophages M1, while showing negative correlations with regulatory T cells (Tregs). (**Figure 9**).

### 3.9 Expression levels of five genes in tissues

**Figure 10:**
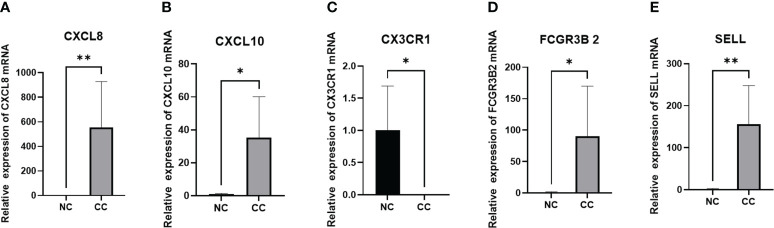
PCR validation of **(A)** CXCL8, **(B)** CXCL10, **(C)** CX3CR1, **(D)** FCGR3B, and **(E)** SELL expression in CC. *P<0.05 **p<0.01

We included 5 cases of chronic cervicitis tissues and 6 cases of CC tissues, in which the expression level of each gene was examined separately. Among them, qPCR was performed for CXCL8, CXCL10, FCGR3B and SELL with GAPDH as an internal reference, and CX3CR1 with 18srRNA as an internal reference. The results showed that the expression levels of CXCL8, CXCL10, FCGR3B and SELL were increased compared to the control. On the contrary, the expression level of CX3CR1 was lower, which was consistent with our prediction (**Figure 10**).

## 4 Discussion

CC is one of the four most prevalent gynecological malignancies, with persistently high incidence and mortality rates in less economically developed countries. Key risk factors for CC including HPV infection, smoking, multiple sexual partners, and HIV positivity. Notably, persistent HPV infection is the primary cause of CC (15, 16). The body’s immune response typically eliminates the inflammation triggered by HPV infection. Chronic inflammation is caused by immune dysregulation and autoimmunity. However, the interaction between HPV infection and the immune microenvironment can foster chronic inflammation, contributing significantly to the process of cervical precancerous lesions to cancer (17). Nevertheless, few studies have focused on biomarkers associated with inflammation and immune cell infiltration in CC. Identifying such biomarkers could directly facilitate disease monitoring and offer new perspectives for targeted CC therapy, early detection and treatment, and enhanced patient prognosis.

Inflammatory responses triggered by HPV infection are known to be determinants of CC development and progression. In this study, we identified 520 inflammation-associated DEGs in CC by integrating two GEO datasets. GO and KEGG enrichment analysis revealed that inflammation-associated DEGs are involved in multiple cellular components, pathways, and diseases. In addition, the key role of the immune response in CC pathogenesis has attracted more attention. Neutrophils, macrophages, B cells, dendritic cells, monocytes, mast cells, and T cells of the immune system can be abnormally infiltrated within CC (18). Therefore, we performed an immune infiltration analysis using CIBERSORT to compare immune cell compositions between CC and normal samples. Moreover, we found three differences in immune cells, with an increase in M1 macrophages and a decrease in resting mast cells and CD4+ memory resting T cells. Furthermore, we employed WGCNA to identify seven immune infiltration-related gene modules. Subsequent correlation analysis revealed that the blue and brown modules exhibited the highest correlation with CC. These findings led to identifying 15 inflammation-associated immune DEGs by crossing inflammation-associated DEGs with significant gene modules. Subsequently, we performed GO and KEGG enrichment analyses. We constructed PPI networks and miRNA-TF-mRNA regulatory networks. Through these comprehensive analyses, we identified five hub genes associated with both inflammation and immune cell infiltration in CC: CXCL8, CXCL10, CX3CR1, FCGR3B, and SELL. Recently, these genes were identified as important factors involved in inflammation and immune response in the physiology and pathology of cancer development.

CXCL8 and CXCL10 belong to the CXC chemokine family and are predominantly secreted by endothelial cells, stromal cells, and immune cells. The primary function of these chemokines lies in directing inflammatory cells to the site of infection and stimulating the release of various growth factors. Notably, these chemokines can also be secreted by tumor cells, interacting with receptors on autologous or other cells within the tumor microenvironment. This dual secretion operates in an autocrine and paracrine manner, exerting a pivotal role in inflammation, neovascularization, and tumour growth and invasion (19–21). Existing research has demonstrated a significant positive correlation between CXCL8 protein expression and CC (22), with HPV infection stimulating CC cells to secrete CXCL10 (23). Our study further validates previous findings that CXCL8 and CXCL10 expression is elevated in cervical cancer.

CX3CR1, the only member of the CX3CR chemokine receptor family, is a seven-transmembrane receptor coupled to a heterotrimeric G protein. It functions as both an adhesion molecule and a chemokine receptor by binding to its ligand, Fractalkine. CX3CR1 is expressed on the surfaces of various cells, for example NK cells and monocytes, and is critical in initiating the immune response. Relevant studies have shown that CX3CR1 is crucial for the recruitment of infiltrating immune cells and is significantly related to clinical stage, histological type, histological grading and distant metastasis, of some tumors (e.g., colorectal cancer, pancreatic cancer) (24–26). However, no studies have investigated its use in the treatment of CC. Based on our findings, we found that CX3CR1 showed reduced expression in cervical cancer.

FCGR3B is a member of the Fcγ receptor family. Not only does it stimulate inflammatory responses, it is also a core immune receptor that controls both humoral and innate immunity, and plays a crucial role in maintaining autoimmune homeostasis and response to infection. Once the balance is disrupted, the individual has an increased susceptibility to autoimmunity and infection (27). Numerous previous studies have suggested that FCGR3B may be a risk factor for a range of autoimmune diseases, such as systemic lupus erythematosus and rheumatoid arthritis (28).The exact rationale for the role of FCGR3B in the treatment of CC is unclear. Yan and his team extracted and sequenced total RNA from 21 CC samples and showed that FCGR3B had high expression levels in these samples (29). This study confirms this observation. Therefore, FCGR3B has the potential to become a biomarker in the diagnosis of CC and a core target for treatment, as well as providing a new direction and reference for the prevention and treatment of CC.

SELL, a member of the selectin family, is a cell surface glycoprotein that induces T-cell homing and enhances T-cells cytotoxicity against tumor cells, thereby exerting anti-tumor effects. Our findings are consistent with previous studies (30). However, the mechanism underlying the role of SELL in the development of CC requires further study. Additionally, we have observed strong diagnostic accuracy for these five indicators in CC diagnosis. This suggests that, as key proteins related to both inflammation and immune mechanisms, they can be used as biomarkers for the early diagnosis and adjuvant therapy of CC.

With the deepening of scientific research, many evidences are supporting the theory that the tumor immune microenvironment influences malignant tumors, and at the same time, tumor-infiltrating immune cells are showing great importance in the detection and development of cancer. Immune factors and immune cells in the tumor immune microenvironment play a central role in tumor development and formation (31). Tumor growth and progression are closely related to the degree of infiltration of immune cells into the tumor, and this relationship may have an impact on the efficacy of chemotherapy and immunotherapy as well as on prognosis (32). In this study, we demonstrated that correlation analysis between CXCL8, CXCL10, CX3CR1, FCGR3B, SELL, and immune cells showed significant associations. Specifically, CXCL8, CXCL10, FCGR3B, and SELL were significantly associated with macrophage M1, whereas CXCL8, CXCL10, CX3CR1, FCGR3B, and SELL were all associated with T cells CD4. In addition, CXCL8, CXCL10, CX3CR1, FCGR3B and SELL were significantly associated with T cells CD4 memory resting. The role of how these five genes affect the immune microenvironment in CC remains unknown. They may alter the tumor immune microenvironment by inhibiting or increasing the occurrence of immune infiltration of specific immune cell subsets of relevance, which ultimately affects the progression of CC. For example, in gastric cancer, CXCL8 promotes an immunosuppressive microenvironment by inducing macrophages (33).Targeting the CXCL8-CXCR2 axis may hinder dendritic cell activation or recruitment, which in turn exerts a critical anti-tumor effect on colorectal cancer (34). From this, we hypothesized that CXCL8, CXCL10, CX3CR1, FCGR3B, and SELL might affect CC progression by regulating the infiltration level of corresponding immune cells. Moreover, detecting the expression level of these genes or the number of specific immune cells as well as their functional status can predict the response to existing therapies, and this prediction can help in the design and optimization of individualized therapy, providing better treatment options for patients and ultimately improving the prognosis of the disease (35). We expect this to provide new directions for future diagnosis and treatment of CC. However, further clinical and experimental studies are needed regarding the complex interactions between these genes and immune cells.

This study had some limitations. This study validated the sample shortage, analyzed the functions of five biomarkers related to inflammation and immune cell infiltration, and performed a prospective analysis. Therefore, expanding the sample size is necessary to further validate the study’s conclusions. In addition, the conclusions drawn herein should be experimentally validated both *in vitro* and *in vivo*.

## 5 Conclusion

In summary, we initially aimed to use various bioinformatic tools and databases to identify useful and potential inflammation-related immune targets associated with CC. Our study showed significant differences in the expression of five genes (CXCL8, CXCL10, CX3CR1, FCGR3B, and SELL) between CC and normal tissues. Various immune cells such as macrophages, CD4+ T cells, and mast cells, may exhibit diverse roles in the progression of CC. Consequently, these cells may become biomarkers for the diagnosis of CC, as well as key treatment targets. Hence, this study provide new ideas and references for the prevention and management of malignant cervical tumors.

## Data availability statement

Publicly available datasets were analyzed in this study. This data can be found here: (GSE39001 and GSE63514) in the Gene Expression Omnibus (GEO)(https://www.ncbi.nlm.nih.gov/geo/).

## Ethics statement

The studies involving humans were approved by the Ethics Committee of the Second Hospital of Shanxi Medical University. The studies were conducted in accordance with the local legislation and institutional requirements. The participants provided their written informed consent to participate in this study.

## Author contributions

WZ: Conceptualization, Data curation, Formal Analysis, Funding acquisition, Methodology, Project administration, Resources, Software, Supervision, Validation, Visualization, Writing – original draft, Writing – review & editing. QL: Conceptualization, Data curation, Formal Analysis, Methodology, Software, Supervision, Validation, Visualization, Writing – original draft, Writing – review & editing. SW: Data curation, Methodology, Supervision, Validation, Writing – review & editing. YL: Data curation, Methodology, Supervision, Writing – review & editing. YB: Data curation, Methodology, Supervision, Writing – review & editing. ZT: Data curation, Methodology, Supervision, Writing – review & editing.
